# Genomic Analysis of *Pasteurella atlantica* Provides Insight on Its Virulence Factors and Phylogeny and Highlights the Potential of Reverse Vaccinology in Aquaculture

**DOI:** 10.3390/microorganisms9061215

**Published:** 2021-06-04

**Authors:** Rebecca Marie Ellul, Panos G. Kalatzis, Cyril Frantzen, Gyri Teien Haugland, Snorre Gulla, Duncan John Colquhoun, Mathias Middelboe, Heidrun Inger Wergeland, Anita Rønneseth

**Affiliations:** 1 Department of Biological Sciences, University of Bergen, N-5006 Bergen, Norway; rebecca.ellul@uib.no (R.M.E.); gyri.haugland@uib.no (G.T.H.); Duncan.Colquhoun@vetinst.no (D.J.C.); heidrun.wergeland@uib.no (H.I.W.); 2 Department of Biology, Marine Biological Section, University of Copenhagen, DK-3000 Helsingør, Denmark; panos.kalatzis@bio.ku.dk (P.G.K.); mmiddelboe@bio.ku.dk (M.M.); 3 STIM/ACD Pharma AS, Karl Johans Gate 16, 0154 Oslo, Norway; cyril.frantzen@stim.no; 4 Norwegian Veterinary Institute, Ullevålsveien 68, P.O. Box 8146 Dep, N-0033 Oslo, Norway; snorre.gulla@vetinst.no

**Keywords:** *Pasteurella atlantica*, lumpsucker, aquaculture, pathogenicity, phylogeny, in silico analysis, virulence factors, mobile elements, vaccine

## Abstract

Pasteurellosis in farmed lumpsuckers, *Cyclopterus lumpus*, has emerged as a serious disease in Norwegian aquaculture in recent years. Genomic characterization of the causative agent is essential in understanding the biology of the bacteria involved and in devising an efficient preventive strategy. The genomes of two clinical *Pasteurella atlantica* isolates were sequenced (≈2.3 Mbp), and phylogenetic analysis confirmed their position as a novel species within the *Pasteurellaceae*. In silico analyses revealed 11 genomic islands and 5 prophages, highlighting the potential of mobile elements as driving forces in the evolution of this species. The previously documented pathogenicity of *P. atlantica* is strongly supported by the current study, and 17 target genes were recognized as putative primary drivers of pathogenicity. The expression level of a predicted vaccine target, an uncharacterized adhesin protein, was significantly increased in both broth culture and following the exposure of *P. atlantica* to lumpsucker head kidney leucocytes. Based on in silico and functional analyses, the strongest gene target candidates will be prioritized in future vaccine development efforts to prevent future pasteurellosis outbreaks.

## 1. Introduction

The prevalence of pasteurellosis in farmed lumpsuckers, *Cyclopterus lumpus*, in Norway has increased in recent years since the first case was recorded in 2012 [1]. Despite a decrease in the number of affected localities [2], *Pasteurella atlantica* [3] remains a significant problem, and as the disease is non-notifiable, under-reporting of outbreaks cannot be excluded. Furthermore, while farmed lumpsuckers are vaccinated against vibriosis and atypical furunculosis, there are no commercially available vaccines against pasteurellosis.

The family *Pasteurellaceae* is composed of commensals, opportunistic- and primary- pathogens and includes the genera *Pasteurella*, *Actinobacillus*, and *Hemophilus* amongst others. The genus *Pasteurella* has a broad host range, but little is known regarding the ecology of marine species. *P. atlantica* isolated from diseased Norwegian lumpsuckers is related to but serologically distinct from *Pasteurella skyensis*, which causes disease in Atlantic salmon (*Salmo salar* L.) in Scotland, and a *P. atlantica* isolate first detected in 2018, which causes disease in Norwegian farmed Atlantic salmon [1,3,4]. Pasteurellosis affects all life stages of a lumpsucker, from fry to fish deployed in salmon cages [4]. As *P. atlantica* has been detected in lumpsucker eggs and milt, vertical transmission may also be possible [5].

In a previous work [6], we showed that whole cell-inactivated bacterin-based vaccines do not provide adequate protection against the disease, despite the high titers of specific antibodies raised. Such a situation highlights the need for a deeper characterization of the bacterium and the identification of immunogenic and protective antigens.

Most pathogenic bacteria have several tools in their genetic arsenal to avoid host defenses and to enhance their survival. Genetic determinants including pathogenicity islands, antibiotic resistance genes, toxins, and adhesins are often shared between bacterial populations via horizontal gene transfer and are commonly associated with plasmids, prophages, and other mobile genetic elements. As adhesins are involved in the early stages of colonization, they can be utilized as targets for vaccine development via reverse vaccinology (RV).

Using in silico bioinformatic analyses, immunogenic antigens for vaccine development can be predicted [7,8]. RV is a rapid process and can reduce vaccine development time by up to 2 years [9]. It is also a more ethical approach due to a reduction in the number of experimental animals required for vaccine testing and results in effective vaccines. In human medicine, RV has been successful in the development of vaccines against several bacterial pathogens reviewed by Sharma et al. 2021 [10]. Pathogens of significant concern such as *Neisseria meningitidis* [11,12], *Mycobacterium tuberculosis* [13], *Chlamydia pneumoniae* [14], *Streptococcus pneumoniae* [15], *Helicobacter pylori* [16], *Porphyromonas gingivalis* [17], and *Bacillus anthracis* [18,19] have been investigated using RV.

RV technologies have improved considerably since the principles were initially developed and applied. The main criteria required for reliable prediction of vaccine targets include subcellular localization, the probability of adhesion functionality, and the number of transmembrane domains [20]. Additionally, using targets that are present only in virulent strains and having protein sequences that are dissimilar to host sequences results in more reliable vaccine targets that also induce strong immune responses [9].

Adhesins are a class of surface-bound proteins involved in facilitating bacterial attachment to host tissues. They are classed as fimbrial or non-fimbrial based on the absence or presence, respectively, of an outer membrane anchor in the protein. Among the different types of adhesins, bacterial lectins are the most common [15]. The mediation of attachment occurs through the recognition of specific carbohydrates, proteins, or lipids presented on the host cell surface.

In addition to attachment, adhesins also promote the delivery of toxins through the upregulation of virulence genes leading to invasion of the host. Furthermore, such interactions can trigger cytokine production or lectinophagocytosis by the host, compounding the severity of disease [21]. For this reason, subunit vaccines that hinder microbial attachment could be of great advantage in combating pasteurellosis in lumpsuckers. Examples of successful adhesin vaccines include those against enterotoxigenic *E. coli* (ETEC) strains in farm animals using the K88 fimbriae [22] as well as uropathogenic *E. coli* (UPEC) known to cause urinary tract infections (UTIs) using the FimCH complex [23]. For the aquaculture industry, vaccines have been tested by targeting major bacterial adhesins from *Aeromonas hydrophila* [24,25], *Vibrio harveyi* [26], and *Edwardsiella tarda* [27].

As research on *P. atlantica* in lumpsuckers is still in its infancy, no commercial vaccines are available against pasteurellosis in lumpsuckers. Simple phylogenetic analyses using the *rpoB* and 16S rRNA genes have shown that NVI-9100 is similar to other *Pasteurella* sp. isolated from lumpsuckers [4]. Whole genome analysis can, however, determine more accurate phylogenetic relationships. The purpose of this study is, therefore, to determine the taxonomic position of the species through whole genome sequencing and to predict potential vaccine antigens via in silico analysis. To further assess the predicted vaccine targets, their expression levels during exposure to lumpsucker leucocytes were analyzed in vitro.

## 2. Materials and Methods

### 2.1. Bacterial Culture, Genome Isolation, and Sequencing

Two *P. atlantica* isolates described in previous studies (NVI 9100 and UiBP1-2013) [4,6,28] collected from clinically sick lumpsuckers were whole-genome sequenced. Briefly, bacteria were grown in tryptic soy broth (TSB) (Becton Dickinson, Sparks, MD, USA) supplemented with 1.5% NaCl and 10% fetal bovine serum, Australian origin, (Gibco, Waltham, MA, USA) at 20 °C with shaking (200 rpm).

Total genomic DNA was isolated using the DNeasy Blood and Tissue Kit (Qiagen, Hilden, Germany) following manufacturer instructions. Briefly, 3 mL of an overnight culture of *P. atlantica* containing a maximum of 2 × 10^9^ cells was centrifuged at 2500× *g* for 15 min and the pellet was resuspended in 180 µL of ATL buffer. Twenty microliters of proteinase K were then added, and the bacteria were incubated at 56 °C in a rotating heat block for 1 h. Two hundred microliters of AL buffer and two hundred microliters of 96% ethanol were added, vortexing well between additions. The mixture was then loaded onto DNeasy Mini Spin columns and washed with the appropriate buffers (AW1 and AW2) prior to elution using buffer AE. The eluted DNA was then purified and measured for concentration before storing at −20 °C until further processing.

Extracted DNA was sequenced using either an Illumina (UiBP1-2013) or PacBio (NVI 9100) platform. Illumina libraries were made using the Nextera DNA Flex Sample Prep kit (Illumina, San Diego, CA, USA) according to the manufacturer instructions and sequenced with Illumina MiSeq (Illumina, San Diego, CA, USA) using V3 chemistry. PacBio libraries were prepared with the Pacific Biosciences 20 kb library preparation protocol, and size was selected using BluePippin (Sage Science, Beverly, MA, USA), with subsequent sequencing performed on a Pacific Biosciences RS II instrument using P4-C2 chemistry and employing three SMRT cells (Pacific Biosciences, Menlo Park, CA, USA).

Raw Illumina sequences were adapter trimmed, quality filtered (Q > 20), de novo assembled using [29,30]. Contigs shorter than 1000 bp or with <5 times coverage were removed from each assembly prior to gene annotation. The genes of the Illumina sequenced genome were predicted by Glimmer 3, version 3.02b; Johns Hopkins University: USA, 1999 [31].

PacBio sequences were de novo assembled using HGAP version 3 (Pacific Biosciences, SMRT Analysis Software version 2.2.0) and circularized with the Minimus2 software version 2 (Amos package). For post-circularization correction of bases, reads were subsequently mapped to the circularized sequences using RS_Resequencing.1 software (SMRT Analysis version version 2.3.0).

This whole genome project has been deposited at GenBank under the Accession Number PRJNA721926 (UiBP1-2013) and Accession Number CP074346 (NVI 9100).

### 2.2. Phylogenetic Analysis

Twenty-three *Pasteurellaceae* genomes (Table 1) were included for phylogenetic analyses.

In order to evaluate similarity among whole genomes, Orthologous Average Nucleotide Identity Tool using OrthoANI [43] was used to provide reliable and fast assessment of average nucleotide identity (ANI) for taxonomic classification purposes. A Similar Genome Finder with high similarity parameters (max hits: 50; *p*-value threshold: 1; distance: 0.5) utilizing Mash, a fast genome distance estimation tool [44], mounted on the Pathosystems Resource Integration Center (PATRIC) platform (https://patricbrc.org/ (accessed on 3 June 2021)) [45,46] was also used to assess the genomic similarity of *P. atlantica* to the bacterial database.

PATRIC [45,46] was used to construct a whole-genome codon-based tree built on 500 single-copy genes [47] present in all genomes studied (Table 1). Both amino acid and nucleotide sequences were aligned using MUSCLE [48] and the codon align function of Biopython [49], respectively. The concatenated alignments of protein and nucleotide sequences were used in order to generate a RAxML method-based phylogenetic tree with branch support values determined by 100 rounds of rapid bootstrapping [50,51]. The tree was visualized using iTOL online platform [52].

### 2.3. Analysis of Genomic Regions and Reverse Vaccinology Approach

The novel genomes were screened against major virulence factor databases. Specifically, the proteome of *P. atlantica* was blasted against VFDB [53] and VICTORS [54] using an e-value of 1 × 10^−10^. A moderate e-value was applied to avoid false positive hits while identifying as many presumptive virulence factors as possible. The prediction was complemented by screening against PATRIC_VF [55], the integrated virulence factor database inside the PATRIC platform (https://patricbrc.org/ (accessed on 3 June 2021)) [45,46]. Subcellular localization for all presumptive virulence factors was examined by PSORTb 3.0 [56] to evaluate putative interaction with the extracellular environment (localization score threshold >7.5). Proteins with both extracellular and outer membrane subcellular localization were imported to SignalP server [57] in order to further assess any known types of signal peptides as well as the pathways in which they are involved during their secretion or anchoring process (threshold >90%). The detection of possible adhesion-related functionality was tested in SPAAN [58]. A Protein Fold Recognition Server, Phyre2 [59], was applied to obtain insights on structure and folding properties of presumptive virulence factors using confidence level of above 97%.

Clusters of genes of probable horizontal origin, known as genomic islands (GIs), were identified by IslandViewer 4, version 4; Simon Fraser University: Canada, 2017 [60]. Inducible prophages were detected using the prophage finder tool, PHASTER [61], while VIRFAM [62] was used to classify intact prophages.

In line with reverse vaccinology principles, the identification of the strongest virulence candidates was deduced through Dynamic Vaxign Analysis, Vaxign [63], a software platform that has been developed and dedicated to vaccine design. The pipeline of Vaxign has integrated PSORTb [56] and SPAAN [58] so it can also be used to validate the previously identified virulence factors. Vaxign-ML [20] was also included in the reverse vaccinology analysis as a machine learning model to improve the prediction of bacterial protective antigens. VaxiJen [64] complemented the analysis to corroborate that the finally selected candidates for vaccine development also can function as protective antigens, which is a prerequisite for vaccine development.

### 2.4. Functional Studies

#### 2.4.1. Processing Bacteria for qPCR Analysis

Cultures of *P. atlantica* (UiBP1-2013) used for the in vitro exposure experiment (Section 2.4.5) were cultivated as described in Section 2.1, harvested in the late exponential growth phase (18 h post inoculation) and centrifuged at 2500× *g* (Beckman Coulter Allegra X-15R) for 15 min at 4 °C. For the gene expression analysis during bacterial cell proliferation in growth medium, 1 mL samples were harvested 14, 16, 18, and 20 h after inoculation. Bacterial cell counts were measured at harvest using a cell counter (CASY Model TT (Innovatis) and CASY worX version 1.26) followed by centrifugation at 4000× *g* (Beckman Coulter Allegra X-15R) for 10 min at 4 °C. For both methods, the growth medium was then discarded, and the samples were stored on ice.

Total RNA was immediately extracted using the Bacterial RNA Kit (E.Z.N.A., Norcross, GA, USA) according to manufacturer instructions. The RNA was then DNase-treated (Sigma-Aldrich, Saint-Louis, MO, USA), converted to cDNA using qScript cDNA Synthesis Kit (Quantabio, Beverly, MA, USA), and stored at −20 °C.

#### 2.4.2. qPCR

Each qPCR reaction contained a volume of 25 μL and consisted of 12.5 μL 2× SYBR Green JumpStart Taq Ready Mix (Thermo-Fisher Scientific, Waltham, MA, USA), 1 μL each of the forward and reverse primers (10 μM final working concentration, Table 2), 0.5 μL of RNase and DNase free water (Sigma-Aldrich), and 10 μL of cDNA (concentration depended on the specific analysis). A C1000 Touch thermal cycler with CFX96 Real-Time System (Bio-Rad, Oslo, Norway) was used for qPCR, with the following cycle conditions: 94 °C for 5 min followed by 40 cycles of 94 °C for 15 s and 60 °C for 1 min. Melting curve analyses were performed after each run (60 to 92 °C at a rate of 1 °C/5 s) to ensure that the specificity of the primers and the qPCR products were visualized on a 2% agarose gel. Three parallel reactions were performed for each gene, and negative controls excluding cDNA (NTC) and cDNA reactions without reverse transcriptase (NRT) were included for all master mixes. The gene expression levels were calculated by the ΔΔCt method [65].

#### 2.4.3. Primer Design and Validation

Genes considered for this work were based on the results from an analysis of genomic regions and potential vaccine targets. The genes selected as reference genes were *rpoD* and *gyrA*. The target gene selected was the putative uncharacterized protein (<Hia>) (Table 2). 

qPCR assays were designed using the software Primer Premier version 6.24 (Premier Biosoft, San Francisco, CA, USA). The five highest rated assays for each target sequence were then chosen for testing. The length of the amplicons was kept between 100 and 250 bp for optimal amplification efficiency. The specificity of the primers was confirmed by qPCR (20 ng of cDNA used in each qPCR reaction), and product size was observed by electrophoresis on 2% agarose gels. All of the qPCR assays produced single amplification products. The best assay for each target gene based on Cq value, non-template controls, melting curves, and the results of electrophoresis were then chosen for further work. The resulting primers used for qPCR are listed in Table 2.

#### 2.4.4. Bacterial Exponential Growth Phase Analysis

*P. atlantica* was grown as described in Section 2.1. At different time points after inoculation, the bacteria were harvested, the RNA was isolated, and cDNA synthesis was performed as described in Section 2.4.1. The synthesized cDNA was diluted across a twofold dilution series to give a range from 10 ng/µL to 0.625 ng/µL of cDNA for both the target and reference genes in qPCR (100 ng to 6.25 ng in each qPCR reaction). The relative gene expression was calculated by the ΔΔCq method using the most suitable dilution from the range tested, and comparisons of gene expression were made to the lowest stable time point of the analysis.

#### 2.4.5. Head Kidney Leucocyte (HKL) Exposure Analysis

Four lumpsuckers were quickly netted and killed by a sharp blow to the head. Leucocytes were isolated from the head kidney on discontinuous Percoll gradients as described previously [66] with the following modifications. The supplemented L-15 medium did not contain gentamicin sulphate since the cells were to be exposed to viable *P. atlantica*. Additionally, resuspension of the isolated leucocytes was performed in L-15 supplemented with 5% fetal calf serum (L-15/FCS). The leucocytes were counted in a CASY-TT Cell Counter TM (Innovatis AG), and the viability (95%) and aggregation factor (1.2) of the cells were determined. The concentration of the isolated leucocytes was then adjusted to 3.3 × 10^6^ cells mL^−1^ in L-15/FCS, and 250 μL volumes were added to each well of the 24-well Nunc plates (approximately 8 × 10^5^ cells per well) (Thermo-Fisher Scientific) and incubated overnight at 15 °C prior to exposure to *P. atlantica*.

A late exponential phase (18 h) culture of *P. atlantica* was prepared as described in Section 2.4.1, re-suspended in L-15/FCS, and adjusted to 1.5 × 10^8^ bacteria mL^−1^. Volumes of 250 μL were then added to each well (approximately 4 × 10^7^ bacteria per well). Sterile L-15/FCS medium was supplied to the leucocytes and used for the non-challenged controls. The cells were then incubated at 15 °C, and samples were collected at 3, 6, 9, 12, and 24 h after exposure. Samples were collected from the wells and centrifuged to remove all media prior to storage at −80 °C, until the total RNA was isolated, DNase treated, and converted to cDNA as described above. For qPCR, 80 ng per reaction was used as the template for both the target and reference genes.

#### 2.4.6. Statistical Analysis

The relative gene expression during the HKL exposure was calculated by the ΔΔCq method [65] and comparisons were made to the negative controls (bacteria without leucocytes). The results were analyzed using two-way ANOVA and Fisher’s LSD for post hoc tests, and differences were considered significant when *p* < 0.05. All statistical analyses were carried out using SigmaPlot version 12 (Systat Software, San Jose, CA, USA).

## 3. Results

### 3.1. Novel Sequenced Genome and Accession Numbers

The length of the PacBio sequenced *P. atlantica* genome (NVI 9100) is 2,301,649 bp, with a GC content of 42.1%, and 2197 coding regions (CDS) were predicted. The length of the Illumina sequenced *P. atlantica* genome (UiBP1-2013) is 2,260,408 bp.

### 3.2. Phylogenomics and Taxonomic Classification

**Figure 1:**
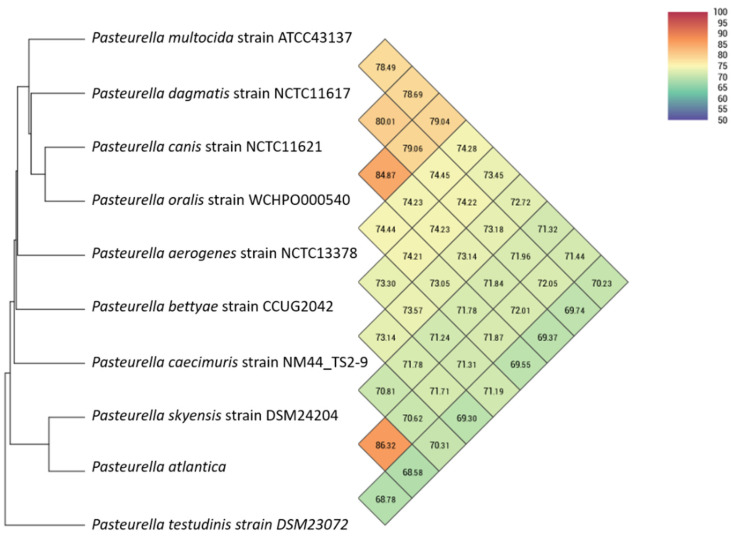
Heatmap generated with OrthoANI values calculated from the Orthologous Average Nucleotide Identity Tool (OAT) software.

The *P. atlantica* strains were isolated from *Cyclopterus lumpus* in a previous study where preliminary phylogenetic analysis was performed based on 16S rRNA and *rpoB* gene sequencing [4]. To assess pairwise similarity percentages among *Pasteurella* species, nine different publicly availably *Pasteurella* species genomes (Table 1) and *P. atlantica* were analyzed using the OrthoANI tool (Figure 1).

When compared to our genome of interest, *P. skyensis* shows the highest OrthoANI values (86.32%) whereas the other species generated much lower values (70 to 72%).

Apart from *Pasteurella* genus, a genomic analysis of *P. atlantica* against the entire publicly available bacterial database, based on whole genome sequences, according to Similar Genome Finder, returned the *P. skyensis* strain DSM 24204 as the only similar genome at a genomic distance of 0.096 (Mash distance threshold: 0.1). The second most similar genome at a genomic distance of 0.145 was the *Ph. uteri* strain NCTC12872, and that was shown only when the threshold increased up to 0.5 (Figure 1). A bigger number of *Pasteurellaceae* family bacteria were found to be less similar, at genomic distances of >0.2, and for the sake of further analysis, representative genomes have been included in Table 1 as well. Substantially low-similarity levels compared to the bacterial database constitute an additional piece of evidence supporting *P. atlantica* as a novel species, knowing that a pairwise Mash distance of at least ≤0.05, approximately corresponding to an ANI of ≥95%, is taken for granted among conspecific bacterial genomes [44].

**Figure 2:**
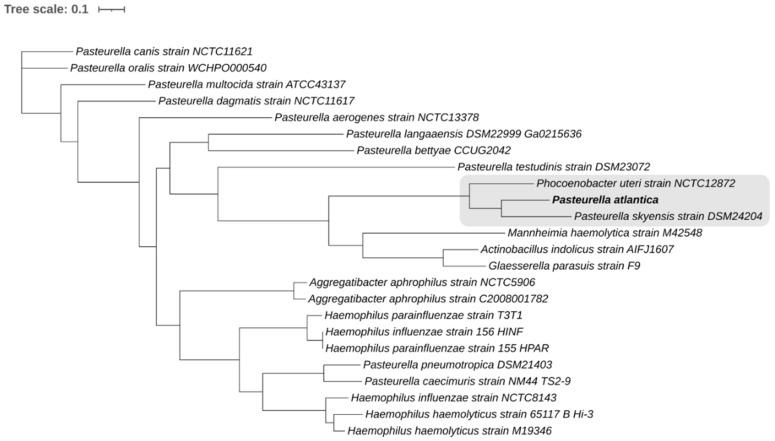
Phylogenetic tree of *P. atlantica* and Table 1 bacterial strains based on compariScheme 500 of randomly selected codons through their whole genome sequences. The monophyletic group in which the novel species belongs is highlighted, while the branch support in all nodes is >98% and has been based on 100 rounds of rapid bootstrapping.

Multiple codons through the entire genomes of Table 1 were used as genetic markers to finalize the taxonomy of *P. atlantica* (Figure 2).

According to the genome-wide analysis, *P. atlantica* clusters together with *P. skyensis* and *Ph. uteri*, forming a robust phylogenetic group supported by 100% confidence level (highlighted in Figure 2). Both monophyletic taxa are phylogenetically distinct since their nodes bear 100% support values.

### 3.3. Genomic Elements

The sequence of *P. atlantica* revealed mobile elements including genomic islands and prophages that should further be analyzed due to their possible role in pathogenicity [67,68,69].

#### 3.3.1. Genomic Islands and Prophages

Bioinformatic analysis on the genome of *P. atlantica* illustrated eleven genomic islands (GIs) (Table 3) with various properties.

GC content of the GIs span between 29.4 and 36.6%. They encompass 207 coding sequences (CDS), approximately 10% of the *P. atlantica* total proteome, and many of them were attributed to virulence-related or broadly proteolytic activity functions. The most prominent putative virulence functions belong to CDS such as RTX-like toxin (GI_1), putative hemolysin toxin of *HlyC* family (GI_1), heme-binding protein (GI_4), bacterial peptidoglycan degrading enzyme of NIpC/P60 (GI_6), spermidine-putrescine binding (GI_8), acetyltransferase of gnat family (GI_9), ctx rstr-like repressor (GI_9), two-partner secretion (TPS) system protein (GI_10), and toxin-antitoxin system proteins (GI_11).

The genome of *P. atlantica* (NVI 9100) harbors five different prophages that were in silico predicted to be intact, hence inducible (Table 4).

P_1 and P_3 were found to be somewhat related to *Enterobacteria* phages, whereas P_2, P_4, and P_5 resemble prophages that have already been described in other *Pasteurellaceae* family bacteria [70,71]. According to BLAST sequence similarity, it was only possible to match 4 out of 26 CDS (7.7%) present in P_1 with any known phage genome implying that this is a quite novel prophage, uniquely present in the genome of *P. atlantica*. Similarly, P_3 seems to be a unique prophage as only 9 of its 49 CDS (18.4%) aligned with *Escherichia* phage D108. On the other hand, P_2 carried 9 of 40 CDS (22.5%) of *Mannheimia* phage vB_MhS_535AP2 while P_4 has homologs of 24 out of 57 CDS (42.1%) that were present in *Mannheimia* phage vB_MhM_587AP1. Last, P_5 has 23 out of 28 CDS (82.1%), resembling *Hemophilus* phage SuMu more, which was also anticipated due to the close phylogenetic relationships between *P. atlantica* and *G. parasuis* [40].

**Figure 3:**
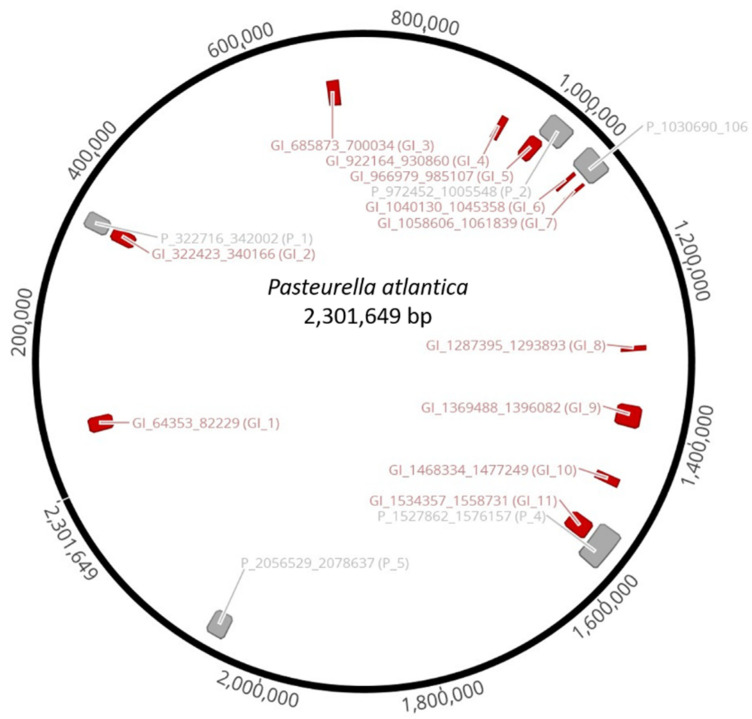
Genomic map of *P. atlantica* that highlights the eleven genomic islands (**red**) and five prophages (**grey**). Five out of eleven GIs are shown to overlap with four out of five prophages.

Interestingly, some of the predicted genomic islands and prophages do overlap in the genome (Figure 3). Specifically, the entire GI_2 is part of P_1, part of GI_5 is included in P_2, both GI_6 and GI_7 belong to P_3, while GI_11 is a part of P_5. Hence, there is a prominent connection between prophages and genomic islands that may be directly linked to the virulence properties of the bacterial host, with the former inciting the mobilization of the latter.

#### 3.3.2. Virulence Factors

**Figure 4:**
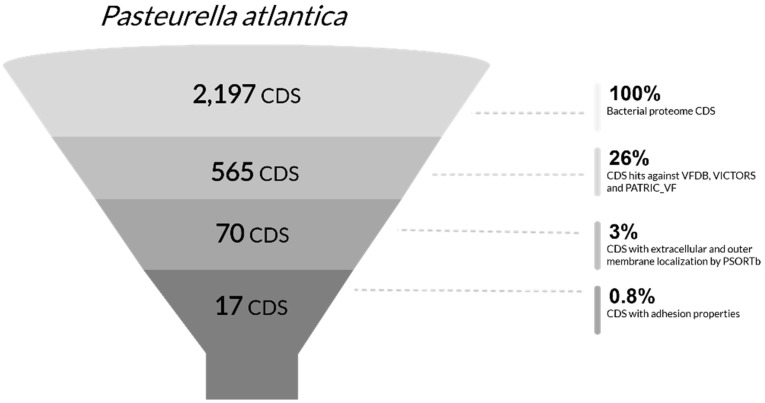
Filtration process of the CDS present in the genome of *P. atlantica* to determine the most likely virulence factors. According to the selection criteria applied during each step of the process, a total of 17 putative virulence factors were predicted as potentially important contributors to the pathogenicity of *P. atlantica*.

The proteome of *P. atlantica* consists of 2197 protein coding sequences (Supplementary Material S1), which were screened against an integrated matrix consisting of the VFDB, VICTORS, and PATRIC_VF databases to generate 565 unique amino acid sequence hits after discarding overlapping hits (Supplementary Material S1). This corresponds to approximately 26% of the 2197 predicted coding sequences. However, significant uncertainty needs to be removed from the analysis to achieve a better focus on the essential virulence factors present in the species. Subcellular localization of presumptive virulence factors showed that approximately 12% (70 out of 565) are presumably either extracellularly secreted (21) or with outer membrane surface exposure (49), setting these proteins as primary candidates for the pathogenicity of *P. atlantica*. The amino acid sequences of the predicted proteins accompanied by their most similar protein sequence GI number (SCL-BLAST), subcellular localization, adhesion, and transmembrane (TM) helices probabilities are reported in Supplementary Material S1. According to their amino acid sequences, 17 out of the 70 virulence factors with extracellular and outer membrane subcellular localization were the most critical for mediating bacterial invasion, such as adhesins with probabilities higher than 75%. Adhesins correspond to 0.8% of the total proteome (Figure 4), and since their expression may be a key determinant of the success of the infection process [72], they could play an imperative role in the pathogenicity of *P. atlantica* (Table 5).

The strongest virulence factor candidate is a novel uncharacterized protein, the function of which would have passed unnoticed without examining its protein structure. Therefore, PDB predictions based on protein fold recognition were included. All PDB predictions reported in Table 5 were above the 97% confidence level and encompassed major virulence components such as hemolysins, toxins, and pili subunits. Specific secretory signal peptides were detected in only 2 out of 17 adhesins, intended to be transported by Sec translocon followed by Signal Peptidase I cleavage (Sec/SPI) to be released to the host.

#### 3.3.3. Vaccine Targets

All 70 CDS with extracellular and outer membrane localization were assessed as potential subunit vaccine candidates. However, apart from adhesion probability, additional factors such as transmembrane helices and orthologue analysis need to be considered for calculating their Vaxign-ML score, which was the metric applied to define their suitability as targets for vaccine development. All vaccine targets with Vaxign-ML scores higher than 99.5% are presented in Table 6, and they are therefore suggested as the most promising vaccine targets. All of the exact scores as well as further details about the remaining 60 extracellular and outer membrane CDS are included in Supplementary Material S1.

Since adhesion probability is not the only criterion for vaccine development, 6 out of the 10 CDS reported in Table 5 are potential virulence factors (Table 6). The list can further be confined by using VaxiJen as a tool to predict the protective antigenic properties of the vaccine targets. Only the putative uncharacterized protein and the Hep/Hag repeat protein had a score >80%, claiming that their antigenicity levels are high enough to trigger the host’s immune response. However, the putative uncharacterized protein CDS not only was ranked first in both tables, scoring the highest Vaxign-ML (100%) and adhesin probability (92.5%), respectively, but also was characterized as a protective antigen, findings that render it the most significant candidate for virulence and potential vaccine development.

According to its amino acid sequence, BLASTP identified it as a YadA-like family protein, a collagen-binding adhesin originally characterized in *Yersinia enterocolitica* [73], that is also present in the genome of *P. skyensis*. However, although the query coverage was 100%, the identity rate was only 58.23%. Based on the predicted structure of the protein, Phyre2 [59] identified it as a membrane protein for cell adhesion similar to Hia (Table 5 and Table 6), which is also a major adhesin expressed by *H. influenzae* [74]. Due to the prominent role that this uncharacterized membrane/cell adhesion protein seems to play in the virulence of *P. atlantica*, it was selected for further evaluation of its expression levels against lumpsucker head kidney leucocytes.

#### 3.3.4. Functional Analysis of the Major Virulence Factor

##### Expression of <*hia*> in Bacterial Exponential Growth Phase

The predetermined time points for measurement were chosen based on growth curves of *P. atlantica* to coincide with the exponential growth phase of the bacteria. Furthermore, from the range of cDNA dilutions tested, 50 ng of cDNA per qPCR reaction yielded the clearest and most consistent results, and this dilution was used to calculate the <*hia*> expression. Out of the two reference genes tested, *gyrA* gave the most stable results compared to *rpoD* (Table 7) and was therefore used to calculate the relative expression of <*hia*> during cell growth and leucocyte exposure studies. This highlights the importance of testing various reference genes for evaluation and stability for expression studies. In this study, although *rpoD* seemed to be a stable gene to use for analysis when primer assays were evaluated, experimental situations proved otherwise.

**Figure 5:**
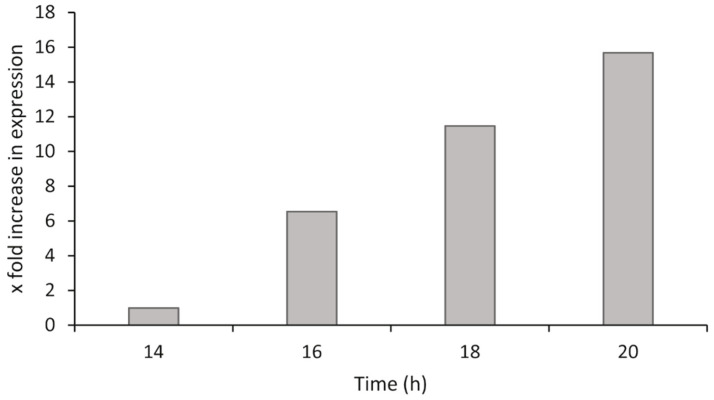
Expression levels of <*hia*> in *P. atlantica* (UiBP1-2013) during growth in liquid medium. The time period corresponds to the exponential growth phase of the bacteria.

The expression level of <*hia*> changed over time, when analyzing samples collected at different time points during the exponential growth phase. The expression levels were upregulated by 6.5×, 11.5×, and 15.7× at 16, 18, and 20 h, respectively, compared to the expression level at 14 h (Figure 5). The expression levels also mirror the exponential increase seen in bacterial numbers from previous work performed on growth curves for *P. atlantica* [28].

##### Head Kidney Leucocyte (HKL) Exposure Analysis

**Figure 6:**
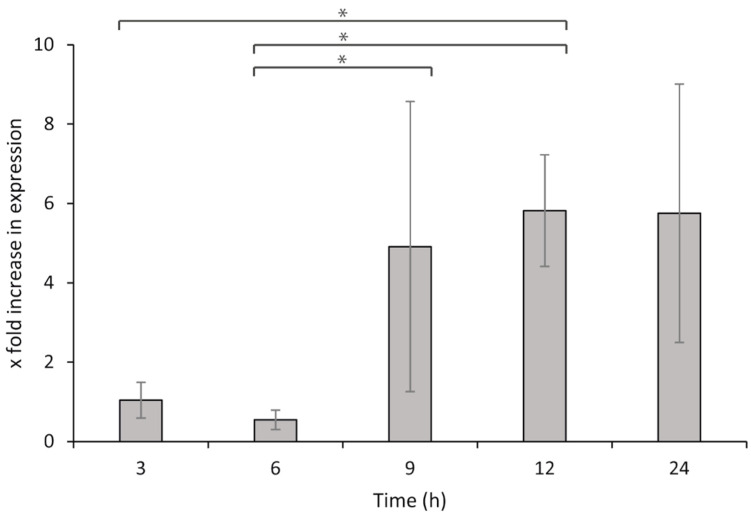
Fold increase in relative expression levels of <*hia*> in *P. atlantica* (UiBP1-2013) over time in the presence of lumpsucker head kidney leucocytes, compared to bacteria not exposed to HKL. Error bars represent standard deviation (*n* = 4). Asterisks represent significant differences in expression between the time points indicated by square brackets.

The expression levels of <*hia*> increased with time, with the highest levels recorded at 9 and 12 h after leucocyte exposure (Figure 6), with significantly elevated expression levels compared with those in the early stages of the exposure (3 and 6 h after exposure). At 24 h after exposure compared to 3 and 6 h after exposure, the bacteria would have entered the lag phase of growth, and the expressions of both the reference and target genes are therefore not comparable with those from earlier time points. Furthermore, statistical analysis also showed that HKL from individual fish did not have an impact on the expression of <*hia*> (*p* = 0.33).

## 4. Discussion

The isolates of *P. atlantica* used in this work were isolated and reported as the causative agent of confirmed outbreaks of pasteurellosis in farmed lumpsuckers from Norway.

### 4.1. Phylogeny Reconstruction

Alarcón et al. [4] carried out preliminary phylogenetic analyses that included the isolates used in this study, using the 16S rRNA and *rpoB* genes, and it was suggested yet not concluded that the isolates might represent a novel species or subspecies. After examining the existing phylogeny of the *Pasteurellaceae* family, which is based on 16S rRNA genes, it can be deduced that using this molecular marker does not sufficiently resolve the true phylogenetic relationships, since it reflects rather incongruent phylogenetic relationships among its members. The available 16S rRNA based phylogenetic tree of the *Pasteurellaceae* family provides clear evidence that the polyphyletic nature prevails in the taxonomy of the family [75] and it has already been proven that there is room for improvement on molecular markers [76]. Despite ongoing research, the current phylogeny of the *Pasteurellaceae* family cannot be considered resolved according to the evolutionary principle of monophyletic taxa formation. Phylogenomic and molecular demarcation studies have been performed both on the major genera of the family such as *Pasteurella*, *Hemophilus*, and *Actinobacillus* [77] and on the reclassification of individual species such as the case of *Hemophilus parasuis* to *G. parasuis* [40].

Hence, the classification of *P. atlantica* in the current study was based on a dataset that includes representative species from both *Pasteurella* and other genera that belong to the *Pasteurellaceae* family to unravel a comprehensive taxonomy for *P. atlantica*. *P. multocida* is the most thoroughly studied member of *Pasteurellaceae* due to its high pathogenicity in a broad range of livestock species [78,79]. The observed orthologous average nucleotide of 86.32% corroborates that *P. atlantica* and *P. skyensis* represent two different species. Whole genome-based phylogeny validated the novelty of the studied genome and its accurate taxonomic placement. Since *P. skyensis* and *Ph. uteri* are the only strains of marine origin out of the entire bacterial database, the habitat seems to be a strong differentiating factor for this taxon of *Pasteurellaceae*. Second, the host species of the bacteria in the marine cluster likely play a role for the speciation process. *P. atlantica* and *P. skyensis* are the closest relatives and both infect marine teleosts [35], whereas *Ph. uteri* isolated from a marine mammal is classified slightly more distant [36]. The major setback in this case is the lack of data in sequenced aquatic *Pasteurellaceae*, a fact that makes it quite precarious to formulate any solid conclusions regarding the trait of virulence. However, the highly emerging incidence and pathogenicity of *Pasteurella* in aquaculture has already brought [4,80] and will definitely bring more genomic data to the scientific community.

### 4.2. Virulence Genes

The scarcity of aquatic *Pasteurella* genomes, particularly from the aquaculture environment, highlights the significance of these *P. atlantica* genomes in exploring genomic elements, including putative virulence factors, antimicrobial resistance genes, and their potential implications for devising precaution strategies such as vaccine development in aquaculture. The abundance of mobile elements such as GIs (11) and prophages (5) in *P. atlantica* further enforces its high pathogenicity that has already been documented in previous studies [4,6,28]. GIs and prophages are very often found in pathogenic bacteria and have consistently been reported as major contributors of virulence factors to their bacterial hosts. Five out of the seventeen strongest candidate virulence factors of *P. atlantica* are in GIs and/or prophages, which in total account for roughly 10% of the genome. This finding is in line with the current literature, which supports the potential of bacterial mobilomes to harbor significant pathogenicity traits. A recent comparative genomic analysis of *Vibrio anguillarum*, a highly virulent aquaculture pathogen, revealed that 64 GIs that belonged to the accessory genome of the species were present in six out of nine of the most virulent strains [67]. In the same study, the role of prophages was also quite prominent, while it was recently proven in another study that marine *Vibrio* harbors a large number of prophage-encoded virulence factors [81]. In the pangenome analysis of *P. multocida*, GIs were also characterized as one of the most important mobile components not only as sources of virulence factors but also as facilitators of foreign genes acquisition. Prophages were again highlighted as core elements of the mobilome, and it was speculated that they may serve as precursors for the formation of GIs [69]. Additionally, facultative intracellular pathogens, such as *P. atlantica* [6,28,82], usually demonstrate quite variable genomes with a fourfold larger genomic content of mobile DNA such as transposable elements and prophages, when compared to obligate intracellular bacteria [83].

Mobile elements are characterized by a high frequency of transfer in the environment through the mechanism of horizontal gene transfer (HGT). HGT can be phage-mediated and can facilitate the dissemination of virulence-associated GIs within and between microbial communities [84], and the presence of a pathogenicity island can potentially define virulence in bacterial pathogens [85]. Apart from sophisticated algorithms [60,61], simpler methods that are based on the distinct GC content of horizontally transferred genes compared to that of the bacterial chromosome have been suggested for the prediction of GIs and prophages [86]. For instance, the pathogenicity islands in *Salmonella typhimurium* (SPIs) have lower GC content than the rest of the bacterial genome [87]. According to the in silico analysis of our study, the average GC content for GIs and prophages were 34.5% and 35.6%, respectively, in both cases lower than 42.1%, which is the average GC content of the *P. atlantica* genome.

Recent progress in in silico analysis tools allowed us to recognize and assess potential virulence factors by exploring the novel genome of *P. atlantica*. Such methodologies constitute fundamental principles of RV, which facilitate vaccine development, while turning extensive, time consuming, and generalized lab work into target-oriented functional analysis and in vitro/in vivo trials. It was not until 2006 when the first standalone RV program designated as NERVE [88] was published, whereas great progress as well as comparative analysis have been achieved and performed in the RV field ever since [89]. The cornerstone of virulence factor prediction has been the determination of subcellular localization of bacterial proteins. Proteins that are extracellularly secreted or exposed on the bacterial surface have good chances to play a role in bacterial virulence.

### 4.3. Adhesins

Adhesins, for instance, which can facilitate the colonization of pathogens to mucosal or other biotic surfaces and which are essential for bacterial pathogenesis and survival may be prominent virulence factors and suitable candidates for vaccine development [72,90]. Screening of the *P. atlantica* genome for gene targets that can be further functionally evaluated as virulence factors generated the 17 strongest candidate genes that may have implications for bacterial virulence, based on subcellular localization and predicted adhesion properties.

Previously published bath, cohabitation, and intramuscular and intraperitoneal challenge trials that included the current *P. atlantica* isolate (UiBP1-2013) illustrated high and acute mortality rates, potentially indicating that extracellular proteins may have a substantial role in the infection dynamics [28], an interim conclusion that is supported by present findings. In our analysis, a putative uncharacterized protein ranked first as both a putative virulence factor and a vaccine candidate.

According to the in silico analysis, the role of this protein was directly linked to major adhesins YadA and Hia in *Yersinia* and *H. influenzae*, respectively, both of which are essential for successful infection of their hosts via human epithelia tissues [73,74,91]. Although their amino acid sequences were not identical, there was a clear connection based on query coverage and confidence level, which in both cases were 100%.

Trimeric autotransporters (TAAs) are a family of non-fimbrial, homotrimeric bacterial adhesins that are secreted by the Type 5c secretion system (T5cSS) and for which the main function is the attachment of pathogenic Gram-negative bacteria to hosts and abiotic surfaces. *Yersinia* adhesin A (YadA) is the most extensively studied member of this family, while others include *H. influenzae* adhesin (Hia) and *Neisseria* adhesin A (NadA).

The type 5c secretion system is of great importance in the development of multivalent recombinant and subunit vaccines, as it enables proteins to be exposed on the bacterial outer membrane, making them potential vaccine targets [92]. This could then also be used as the basis for further work to elucidate the structure of the Hia-like protein investigated in the current study.

YadA showcases the invasive nature of *Y. enterolitica* and *Y. pseudotuberculosis*, which colonize the intestine by adhering to the intestinal mucosa [93]. Immunologically, YadA has been shown to be a highly immunogenic antigen. In fact, Tahir and Skurnik (2001) [93] found that immunizing mice with live bacteria raised antibodies against only epitopes located in the N-termini, whereas using inactivated bacteria raised antibodies against other epitopes as well. In addition, a study [94] found that mice immunized using a recombinant YadA vaccine had higher survival compared to those vaccinated with inactivated *Y. pseudotuberculosis*.

Hia is an adhesin found in 25% of clinical non-typable strains of *H. influenzae* (NTHi). It mediates adherence to host cells and causes various diseases in humans, including respiratory tract infections and meningitis [95]. Hia differs slightly from other TAAs and is thought to represent a new sub-family. It too is highly immunogenic, however, and antibodies were strongly induced following natural infection observed in humans. Furthermore, opsonophagocytic antibodies were generated in serum in guinea pigs and mice [96]. However, due to its prevalence in so few strains and despite its high antigenicity, it cannot be considered a vaccine target on its own but in combination with High Molecular Weight adhesin 1 (HMW1), High Molecular Weight adhesin 2 (HMW2), and outer membrane vesicles (OMVs). In this way, a vaccine containing the three adhesins covers 95% of NTHi and confers better protection [97].

The significantly higher expression levels of <*hia*> detected in the current study following in vitro exposure to lumpsucker leucocytes after 9 and 12 h together with the in silico results strengthen its candidacy as both a major virulence factor and a potential target for subunit protein antigen, mRNA, or DNA vaccine development.

The expression of <*hia*> in the absence of a host correlates with previously observed increases in bacterial growth rates [28] and indicates that the adhesin is typically expressed during bacterial growth. In the exposure experiment, bacteria were harvested from the growth medium at 18 h after inoculation when both bacterial growth and <*hia*> expression were at peak levels and combined with HKL in cell culture medium, which does not favor bacterial growth. The results from this experiment then demonstrate that the expression of <*hia*> increases significantly in the presence of the host, following an initial period of acclimation (up to 6 h, as seen in Figure 6). This implies that the presence of host leucocytes serves as a trigger for the bacteria to significantly increase the expression of adhesins (between 6 and 9 h after exposure) and shows that the increased expression is due to the presence of the host and not an increase in bacterial cell numbers. Furthermore, it indicates bacterial cell attachment to leucocytes as an initial step towards infection, as previously suggested by Ellul et al. (2019) [6].

### 4.4. Vaccine Development

Vaccines targeting adhesins can confer protection by two major mechanisms, one of which is by inducing neutralizing antibodies, which attach to adhesins and prevent attachment and subsequent infection. The other involves inducing opsonizing antibodies, which attach to adhesins and increase recognition of the pathogen by Fc receptors on phagocytes, leading to phagocytosis, or by activation of the complement system [98], as shown by Winter and Barenkamp [96] in their experiments with Hia.

Ellul et al. [6] recently assessed the efficacy of formalin-inactivated *P. atlantica* vaccine in lumpsucker, and although immune response and antibody production were prominent, the protection levels provided by the vaccine were relatively weak. Inactivated bacterin is the most common and traditional type of vaccine used in aquaculture; however, alternatives such as subunit recombinant, mRNA, or DNA vaccines may be the next step in solving the problem [99]. This is especially the case with the recent advances in human mRNA vaccines against SARS-CoV-2, which point to potential avenues for fish vaccines. These vaccines can be developed using various techniques, including harvesting and purifying the target directly from the pathogen, or using recombinant expression vectors, such as *E. coli* [99]. Pending further work, the former method could be a possible step forward in the case of *P. atlantica* as high expression rates were observed in bacteria in their exponential growth stage.

Some of the most successful recombinant protein subunit vaccines in the aquaculture industry include those against IPN (MSD Animal Health) and ISA in Chile (Centrovet). DNA vaccines have also been produced for use in Atlantic salmon against IHN in Canada and against SPD in Norway (Elanco). Subunit vaccines against the bacterial fish disease furunculosis in *Oncorhynchus mykiss* [100] have been tested. All targets for the vaccine development were identified in silico, employing the RV principles and found to be protective. Recombinant protein production has already been used in order to protect farmed fish against infections by *V. anguillarum*, *Edwardsiella tarda, Aeromonas hydrophila*, and *Francisella orientalis* [101] in all cases leading to the suggestion of potential vaccine candidates [102,103,104]. Following these lines, our study highlights the potential that in silico analysis may have in the future formulation of a novel vaccine against pasteurellosis.

Commercial vaccines are available only for a few infectious diseases in aquaculture, and given that the needs for fish and shellfish protein as well as the number of cultured aquatic species are constantly increasing, novel vaccine development will be essential to guarantee improved animal welfare and a safe, sustainable, and healthy product.

## 5. Conclusions

In this study, we report the first fully sequenced genomes of *P. atlantica*, an emerging pathogen in *Cyclopterus lumpus* in Norwegian and Scottish aquaculture. The wide phylogenetic distance from the closest bacterial relative, *P. skyensis*, and the very few available *Pasteurellaceae* members of aquatic origin highlight the need for intensification of sequencing efforts that should be focused on *Pasteurella* strains from the aquaculture environment. In silico genomic analysis revealed numerous target genes that may be responsible for the virulence of this species, and a major role was attributed to mobile elements such as prophages and GIs. Functional analyses showed that the expression levels of an uncharacterized protein increased significantly when *P. atlantica* was exposed to leucocytes from lumpsuckers, complementing the in silico analysis by designating a prospective virulence gene and by promising candidate for vaccine development. Further assessment of virulence in larger scale as well as formulation of experimental vaccines will shed more light on devising a precautionary strategy against a serious emerging disease in aquaculture.

## Figures and Tables

**Table 1:** Twenty-three *Pasteurellaceae* genomes used to evaluate the phylogenetic position of *P. atlantica*.

Bacterial Species Name (GenBank)	Animal Host	Country	Genome	Ref.
*Pasteurella* strains				
*Pasteurella multocida* strain ATCC43137	Pig	n/a	Assembly	NCBI
*Pasteurella dagmatis* strain NCTC11617	Human	n/a	Assembly	[32]
*Pasteurella testudinis* strain DSM23072	Desert tortoise	USA	WGS—69 contigs	[33]
*Pasteurella bettyae* strain CCUG2042	Human	USA	WGS—47 contigs	NCBI
*Pasteurella langaaensis* strain DSM22999 Ga0215636	Chicken	Denmark	WGS—29 contigs	[32]
*Pasteurella caecimuris* strain NM44_TS2-9	Mouse	Canada	WGS—53 contigs	NCBI
*Pasteurella canis* strain NCTC11621	Human	n/a	WGS—20 contigs	[32]
*Pasteurella oralis* strain WCHPO000540	Human	China	WGS—30 contigs	[34]
*Pasteurella skyensis* strain DSM24204	Atlantic salmon	Scotland	WGS—56 contigs	[35]
*[Pasteurella] aerogenes* strain NCTC13378	n/a	Finland	Assembly	NCBI
*Pasteurella pneumonotropica* strain DSM21403	Mouse	USA	WGS—22 contigs	NCBI
Non-*Pasteurella* strains				
*Phocoenobacter uteri* strain NCTC12872	Harbor porpoise	Scotland	WGS—5 contigs	[36]
*Hemophilus hemolyticus* strain 65117 B Hi-3	Human	Australia	WGS—16 contigs	NCBI
*Hemophilus hemolyticus* strain M19346	Human	USA	Assembly	[37]
*Hemophilus influenzae* strain 156_HINF	Human	USA	WGS—41 contigs	[38]
*Hemophilus influenzae* strain NCTC8143	Human	UK	Assembly	NCBI
*Hemophilus parainfluenzae* strain 155_HPAR	Human	USA	WGS—61 contigs	[38]
*Hemophilus parainfluenzae* strain T3T1	n/a	n/a	Assembly	NCBI
*Aggregatibacter aphrophilus* strain C2008001782	Human	USA	WGS—16 contigs	[39]
*Aggregatibacter aphrophilus* ATCC33389 strain NCTC5906	n/a	UK	Assembly	NCBI
*Glaesserella parasuis* strain F9	Pig	Spain	WGS—182 contigs	[40]
*Actinobacillus indolicus* strain AIFJ1607	Pig	China	Assembly	[41]
*Mannheimia hemolytica* strain M42548	Ruminants	n/a	Assembly	[42]

**Table 2:** Details of primers for reference genes and the target gene used for qPCR.

Gene	Primer Name	Sequence 5′-3′	Primer Length (bp)
*gyrA*	#27-B_GYRA_F3	GTTCATCGGGTATTGCGGTCGGTAT	25
	#28-B_GYRA_R3	TCCTGTGCGGTAAGCGTCTTCG	22
*rpoD*	#46-B_RPOD_F1	GGACGTGATGCGACACCTGAAGAAT	25
	#47-B_RPOD_R1	AGTGGCTGTGCAAGTGCAGTATCTT	25
Putative uncharacterized protein <Hia>	#70-B_HIA_F4	AGGTGTGGGTTCATTCGCTGTGG	23
	#68-B_HIA_R2	CCGATTGCTGCCGCTTGTTGTTC	23

**Table 3:** Genomic features of the eleven genomic islands that were found in the genome of *P. atlantica* (NVI 9100).

#	Sequence Location	Sequence Length (bp)	GC%	CDS
GI_1	GI_64353_82229	17,877	31.3	19
GI_2	GI_322423_340166	17,744	34.8	26
GI_3	GI_685873_700034	14,162	34.4	11
GI_4	GI_922164_930860	8697	36.6	12
GI_5	GI_966979_985107	18,129	34.7	26
GI_6	GI_1040130_1045358	5229	35.2	7
GI_7	GI_1058606_1061839	3234	33	9
GI_8	GI_1287395_1293893	6499	29.4	7
GI_9	GI_1369488_1396082	26,595	31.1	34
GI_10	GI_1468334_1477249	8916	29.7	12
GI_11	GI_1534357_1558731	24,375	35	44

**Table 4:** Genomic features of the five prophages that were found in the genome of *P. atlantica* (NVI 9100).

#	Sequence Location	Sequence Length (bp)	GC%	CDS	Most Closely Related Phage	Predicted Family	NCBI Accession Number
P_1	P_322716_342002	19,287	35	26	*Enterobacteria* phage P4	Unknown	NC_001609
P_2	P_972452_1005548	33,097	37.3	40	*Mannheimia* phage vB_MhS_535AP2	Siphoviridae	NC_028853
P_3	P_1030690_1066158	35,479	35.6	49	*Escherichia* phage D108	Siphoviridae	NC_013594
P_4	P_1527862_1576157	48,296	35.2	57	*Mannheimia* phage vB_MhM_587AP1	Myoviridae	NC_028898
P_5	P_2056529_2078637	22,109	35	28	*Hemophilus* phage SuMu	Myoviridae	NC_019455

**Table 5:** Seven extracellular and ten outer membrane localized putative virulence factors of the genome of *P. atlantica* (NVI 9100). E: extracellular, OM: outer membrane, GI: Genomic island, P: Prophage, and ND: Not designated.

#	CDS	Localization (%)	Adhesin (%)	Signal Peptides	Protein Data Bank (PDB) Header (PDB Molecule)	Designated Genomic Area
1	Putative uncharacterized protein <Hia>	E (96)	92.5	n/a	Membrane protein/cell adhesion (Hia)	ND
2	Uncharacterized protein	E (96)	74.8	n/a	De novo protein (designed helical bundle)	GI_6 and P_3
3	Hemolysin-type calcium-binding region	E (96)	82	n/a	Cell adhesion (surface associated protein csha)	ND
4	Putative collagen triple helix repeat protein	E (96)	86.2	n/a	Membrane protein/cell adhesion (Hia)	GI_3
5	HbP1 protein	E (97)	81.5	n/a	Structural protein/contractile protein (collagen I alpha 1)	GI_2 and P_1
6	Uncharacterized protein	E (97)	76	Sec/SPI	Metal transport (hemophore)	ND
7	Pilus A	E (96)	82.9	n/a	Cell adhesion (fimbrial protein)	ND
8	Hep/Hag repeat protein	OM (95)	85.8	n/a	Cell adhesion (hep_hag family)	ND
9	Uncharacterized protein	OM (89)	82.6	n/a	Toxin (hemolysin)	ND
10	Protein PfhB1	OM (99)	75.4	n/a	Membrane protein (znud)	ND
11	Putative uncharacterized protein 4	OM (100)	74.7	n/a	Toxin (hemolysin)	ND
12	Putative septum site-determining protein MinC	OM (99)	82	n/a	Pili subunits (pili subunits)	ND
13	Putative uncharacterized protein 26	OM (95)	86.3	Sec/SPI	Pili subunits (pili subunits)	P_5
14	Phage-related protein tail component-like protein	OM (95)	74.6	n/a	Signaling protein receptor (interferon alpha beta receptor 1)	P_2
15	Type IV pilus biogenesis/stability protein PilW	OM (99)	80	n/a	Transferase (udp-n-acetylglucosamine--peptide n-)	ND
16	Auto transporter beta-domain protein	OM (95)	84.8	n/a	Hydrolase (esterase esta)	ND
17	Outer membrane antigenic lipoprotein B	OM (99)	79.7	n/a	Sugar binding protein (chitin elicitor-binding protein)	ND

**Table 6:** Three extracellular and seven outer membrane localized proteins are the most promising candidates for vaccine development against *P. atlantica* (NVI 9100 and UiBP1-2013). E: extracellular, OM: outer membrane, GI: Genomic island, P: Prophage, and ND: Not designated.

#	CDS	Localization (%)	Adhesin (%)	Signal Peptides	PDB Header (PDB Molecule)
1	Putative uncharacterized protein <Hia>	E (96)	92.5	n/a	Membrane protein/cell adhesion (Hia)
2	Uncharacterized protein	E (96)	74.8	n/a	De novo protein (designed helical bundle)
3	Uncharacterized protein	OM (89)	82.6	n/a	Toxin (hemolysin)
4	Hep/Hag repeat protein	OM (95)	85.8	n/a	Cell adhesion (hep_hag family)
5	Protein PfhB1	OM (99)	75.4	n/a	Membrane protein (znud)
6	Putative uncharacterized protein 4	OM (100)	74.7	n/a	Toxin (hemolysin)
7	Serine protease sat autotransporter	E (84)	69.4	Sec/SPI	Hydrolase (hemoglobin protease)
8	PfhB1	OM (100)	69.5	n/a	Toxin (hemolysin)
9	Uncharacterized protein	OM (100)	63.3	Sec/SPI	Transport protein (translocation and assembly module tama)
10	Outer membrane protein assembly factor BamA	OM (100)	50.8	Sec/SPI	Membrane protein (outer membrane protein assembly factor BamA)

**Table 7:** Reference and target gene primers qPCR assay performance.

Gene	Amplicon Size (bp)	Assay Efficiency (%)	Correlation (R^2^)
*gyrA*	200	102	0.998
*rpoD*	173	104	0.987
Putative uncharacterized protein <Hia>	192	96	0.984

## Data Availability

This whole genome project has been deposited at GenBank under the Accession Number PRJNA721926 for isolate UiBP1-2013 and accession number CP074346 for isolate NVI 9100.

## Supplementary Materials

The following are available online at https://www.mdpi.com/article/10.3390/microorganisms9061215/s1, Supplementary Material S1: Database with an extensive description of the sequences from the proteome-filtering process (virulence factors and adhesins)
